# Professionals' Experiences of the Relations between Personal History and Professional Role

**DOI:** 10.1155/2013/265247

**Published:** 2013-03-26

**Authors:** Hege Sjølie, Bengt Karlsson, Per-Einar Binder

**Affiliations:** ^1^Faculty of Health Sciences, The University College of Buskerud, Box 7053, 3007 Drammen, Norway; ^2^Institute for Research in Mental Health and Substance Abuse, Faculty of Health Sciences, The University College of Buskerud, Box 7053, 3007 Drammen, Norway; ^3^Department of Clinical Psychology, The University of Bergen, Box 7807, 5020 Bergen, Norway

## Abstract

The purpose of this paper is to explore whether and how workers in a crisis resolution home treatment (CRHT) team experience the relationship between their personal history and professional role. This paper is based on 13 in-depth interviews with health professionals working in CRHT. The interviews were analysed using a hermeneutic-phenomenological approach. Participants expressed that there is a relationship between their personal history and professional role, and three themes are highlighted as particularly important in, namely experiences related to the participants as individuals, work-related experiences and family-related experiences. The participants write meaning into the relationship between their personal history and professional role. By relating and exploring their own life stories in the interviews, they work on forming meaning and identity.

## 1. Introduction

Crisis resolution home treatment (CRHT) is a work approach that may put high emotional, relational, and professional demands on the team members. It challenges some traditional professional roles within the mental health care field by moving crisis resolution away from in-patient care and into the patients' home. In most western countries, the services for people with mental health problems have gone through major changes over the last decades [1–3]. Norway, like many other western countries, is experiencing a rapid transition from traditional institutional care to an expansion into community-based mental health care for people with mental health problems [4].

CRHT team workers' experience of their personal background and how they think it may contribute to their interest and motivation for their work roles as professionals is relatively little explored. The following research question will be explored and discussed. 

How do CRHT team members experience possible relations between their personal histories and professional roles? 

A professional role consists of experiences based on working background, education, career [5–9], and personal experiences [5, 7]. At the same time, the construction of a personal self is closely related to one's history, family background, and life experiences, including the narrative organization of everyday life and specific events that are memorable to them [10, 11]. If and how a professional role as a CRHT team member and the experience of one's personal self may be interrelated is an interesting question. The aim of this paper is to gain a deeper understanding by exploring how the team members reflect upon the experiences of their own personal histories and professional roles throughout life and any possible relationship between these.

### 1.1. The Development and Characteristics of CRHT Teams

Crisis resolution home treatment (CRHT) team is a significant recent development within the home-based mental health services. These teams provide an alternative to in-patient acute care services, offering assessment as well as direct care [4, 12]. Models of community care are being established that aim to minimize hospitalisation and maximize acute care and rehabilitation within the context of family and immediate social environment of individuals [13]. The development of community mental health services is in line with the policy mandates of the World Health Organization and European policy [2, 3, 14–16]. 

The key service characteristics of CRHT teams include (a) timely response services to individuals in crisis, (b) provision of assessment of service-users in the context of home and family, (c) determination of a course of action from among a range of options that include hospitalization, home treatment, crisis resolution by the team, and next-level referrals to health or social services, and (d) provision of mental health care as deemed necessary in the context of home and family [12]. The functions and characteristics of CRHT teams vary, but typically they emphasize being multidisciplinary, a low patient-staff ratio, rapid assessment, short-term care (4–6 weeks), and attention both to clinical and social needs [17]. 

The literature provides some insights into the effectiveness of CRHT teams both at macro and individual levels. Established CRHT teams in England have been shown to reduce hospitalization. Glover et al. [18] report that teams providing a full 24-hour seven-day-a-week service reduce hospital admission on average by 23%, whilst teams without “full cover” also reduce admission by on average 10% in comparison with areas without these services. There are also other reported benefits including user satisfaction and family engagement. For example Hultberg and Karlsson [19] note that the service users of a CRHT team felt a greater sense of control and appreciated having choices and opportunities for participation.

### 1.2. Context and Partners

This paper is based on a four-year research project where the main project consisted of three different studies, exploring crisis resolution and home treatment in the community mental health service. The specific aim of the main project was to develop and advance comprehensive knowledge about the workers of CRHT teams from three perspectives. The first study focused on the development of a CRHT team, examining the discourses and practice patterns of a CRHT team and the experiences of the team members. The second study focused on the experiences of CRHT service users, and the third study focused on examining aggregate-level outcomes in relation to the characteristics of CRHT teams that was in place across mental health centers in Norway. The research context was the practice of team members of a local CRHT team, which started in this model of service in 2007. The team members as active participants in this participatory action research were involved in an ongoing process of developing their practice in the new service model through practice and research. This paper is part of study one: the focus is on how professional practitioners establish meaning in their work on background of the narratives about their personal interests and motivations. 

### 1.3. Background

Previous studies have focused on the development of work-related identity and expertise within traditional professional health care roles. One is the area of training of health professionals, where previous experience within the work role is explored in connection to skilled reflection and learning [6]. Development of professional identity in medical education has also been studied with regard to consolidation of core identity, development within embodied individuals, interactional aspects of identity, and institutional aspects of identity [8, 9]. These studies concern the topic of how work experiences, role, and identity are related to training or education. Other studies raise questions of how personal lived experiences are integrated as part of professional roles [5, 7]. This touches upon the importance of reflection. In our paper, we focus on the relationship between the “personal” and “the professional identity,” and we do so with a relatively new and nontraditional work role within the mental health field as a point of departure: the experience of being a person that has found a role that fit him or her as a member of a CRHT team. 

## 2. Method

### 2.1. Methodological Approach

A hermeneutic-phenomenological approach was chosen to explore the first-person experiences of the team members in terms of their personal histories and professional roles. The aim is to get the participants' descriptions of whether and how they experience the relationship between their personal histories and professional roles. Hermeneutics is based on an understanding that formation of knowledge in one way or another always includes a form of interpretation [20, 21] and phenomenology is based on an intention to explore and describe meaning pattern in experiences as they are understood and told by the participants [22]. All formation of knowledge takes place in a context where preconceptions make understanding possible [23], and these are unavoidable parts of our interpretation of the material. When interpreting we try to stay as close as possible to the stories as told by the participants, and as much as possible become self-reflexive on our preconceptions. Based on a reflexive methodology, there is a reflection on the researchers' prior understanding of the participants' personal histories and professional roles, and also on how this prior understanding might have influenced the analysis of the material [24–26]. By hearing and interpreting the stories a meaning was created, arising from the participant's context, the interview context and from the understanding of the researchers. The stories are reflected on with the participant during the interview, and also by the researcher when rereading the interviews. 

### 2.2. Participants

There are 13 participants in the study, 11 women and two men, aged between 27 and 59. Their professional backgrounds are multidisciplinary, consisting of eight nurses, three social workers, and two psychologists. All nurses and social workers have completed one or more extended courses related to mental health issues such as psychiatric nursing, cognitive therapy, or family therapy. All but one psychologist and one nurse have attended a course of training in CRHT. And all but the two psychologists have more than two years of work experience related to mental health, both hospital and community based.

### 2.3. Data Collection

The individual interviews were conducted during a two-month period from mid-October to mid December 2009. The exception was an interview with one of the psychologists who started work in autumn 2010: this took place in December 2010. Two of the authors were directly involved in collecting data. The first author conducted eight interviews and the second author conducted five. Each interview lasted between 45 and 90 minutes. Most of the interviews were held at the team base, but one was held at the researchers' office. 

### 2.4. Interview Protocol

The interviews were semistructured and based on an interview protocol with a few open questions, beginning with the questions “what do you think is important in your own personal history and professional role?” and “In what way do you see a relationship between your own personal history and professional role?”. Then followed the question “are there any special circumstances relating to your personal or professional history that make you work in this team?” to elaborate upon the answers the participants gave on how they experienced connections. The last questions probed what the participants thought about their own professional development, if there were special professional issues that interested them and if they had any thoughts on why that was so. The interview was conducted as a dialogue and a mutual exploration of the participants' narratives was emphasized. All questions in the protocol were asked in all interviews, but not necessarily in the order mentioned above. All interviews were taped and transcribed.

### 2.5. Data Analysis

The analyses were conducted using the following steps, informed by the models for qualitative data analysis described by Kvale and Brinkmann [20], Van Manen [22], Finlay [24, 25], Binder et al. [21, 27], and Malterud [28, 29].

- The first and second author noted their initial impressions after conducting the individual interviews and discussed these observations to develop a preliminary understanding of some basic patterns of both heterogeneity and homogeneity within the participants experiences expressed in the material.
- The first author sorted out the parts from the transcribed material that were relevant for the research questions. All researchers read edited parts of the transcribed material to obtain a basic sense of the participants' experiences [29]. An emerging recognition of some of the researchers' personal and professional preconceptions was also part of this phase.
- The first author identified separable content units that represented different aspects of the participants' experiences. The content units are citations from the participants' experiences that all together form a statement.
- The first author developed “meaning codes” for those units, which are concepts or keywords attached to a text segment in order to permit its later retrieval [20]. The concepts or keywords reflect the researchers' interpretation of the theme. Then the first author edited the text in accordance with those codes into coded groups of text.
- The first author summarized the meaning within each of the coded groups of content units, the meaning codes, into conceptions and overall descriptions of meaning patterns reflecting what, according to our understanding, emerged as the most important aspects of the participant's experience. The knowledge and meaning of each code are interpreted and condensed, presenting the nuances of each code in an experience near language as overall descriptions. The steps in this process might be illustrated as in Table 1.
- All three authors turned back to the edited parts of the transcribed material, the citations from the interviews, to check whether voices and points of view should be added, could develop the descriptions of themes further, or represented correctives to the preliminary line of interpretation. The third author, who was not part of the team of interviewers, had a leading role in critically auditing the identification of themes. This led to a reorganization of some of the categories that the third author brought back to the others for consensual discussions.
- The content units and meaning codes identified and agreed by all three authors were summed up. Based on the conceptions, overall descriptions and the chosen quotes from the data, patterns are described and explored. This process gave a list of themes, and a view on how the CRHT workers experience and describe the relationship between their personal stories and professional roles.

## 3. Findings

When analyzing the individual interviews the strongest links we found between personal history and professional roles were in the areas of; (1) experiences related to the participant as an individual, (2) work-related experiences, and (3) family-related experiences, as illustrated in Table 2. (1) Experiences related to the participant as an individual consist of (a) personal qualities and (b) personal interests. (2) Work-related experiences can be described as profound personal experiences in a work context contributing to the way one handle the work role as CRHT member. (3) Family-related experiences are divided into experiences with (a) family members with mental health problems and (b) family members working in mental health services. These themes from the participants' personal histories are experienced to be connected to their professional stories.

The way we see personal history is inspired by Schütz concept of life-world [30] and McAdams models of how biography is structured [10]. These have elements of a natural attitude within the person, but also experiences from life which all together forms the individuals' life-world. We understand the personal history as consisting of a temporal sequencing of formative events in one's life, interactions with important persons and how this gave rise to certain characteristics of oneself. This character might be a parallel to what Schütz call a natural attitude. Based on these characteristics of oneself as a person, the personal history is formed by personal interests developed, work-related experiences, and family experiences with mental health problems or work in mental health services. The personal history is based on the characteristics of oneself as a person and evolves through the personal interests and experiences related to work and family life.

### 3.1. The Relationship between Characteristics of Oneself as a Person and the Chosen Work Role: Quick, Safe, and Creative

The characteristics of oneself as a person are part of the individuals' natural attitude [10, 30]. It forms a basis within the person, holding characteristics that make the person fit for certain tasks and situations. 12 participants gave description of personal qualities that were significant for their work, and many of them related these qualities to the history of their development into their professional role as a CRHT member. The personal characteristics as part of their personal history were connected to their professional role by describing how the characteristics fitted or was necessary for them to fulfill their work tasks in the CRHT team.

Ability to make contact was seen as an important personal characteristic by the team members. One participant describes how the ability to establish relationships quickly was an important personal quality for her work role.

*I am good at establishing relations quickly. I make close connections with people fast. I can endure pretty intense relationships for a short period of time, but not for long.*

This refers to an ability to establish deep emotional relationships with the patient relatively quickly—to get relatively close relatively fast. In a service where one meets with patients for a short period of time, this ability was seen as valuable. This ability to establish deep emotional relationships fast and keep them for a short period of time is a personal characteristic that forms a basis for her personal history. In relation to a work situation in a CRHT team moving in fast, doing quick assessments and closing the case this personal characteristic is essential for being able to help the patient assigned. It contributed an essential element to her professional role in the CRHT team. Being able to establish deep relations quickly is a thematic component that relates to personal characteristics. 

Feeling safe oneself was described as an important quality by some participants. It was related to feeling safe in stressful and dramatic work situations. The ability not to be easily scared was emphasized, as was the importance of feeling confident with colleges when working in home treatment.

*I can be scared, but I cannot remember that that has ever happened when with a patient.*

This feeling of safety was connected to professional experience. When gaining experience in working with an acute mental health crisis, a participant described how the feeling of safety could be learned.

*I can see how younger colleagues talk to the doctor more often because they feel unsafe regarding the patients. That is a way that they can gain experience and confidence.*

Two participants connected this learning to how experience can make one safer and more relaxed by knowing one's personal potential to solve difficult situations in situ even without having a solution ready for every situation. This was seen as personal maturation from experience in the field. 

Another personal quality described as important was the ability to make patients feel safe. This quality was often connected to professional aspects of crisis resolution as acting out, or how to cope when working with suicidal patients. For the patient, this was connected to presentation, not necessarily the words themselves but how they were presented.

*I am not so good at talking, finding the right words. But short and clear speech suits me well. Short sentences, loud and clear, that cannot be misunderstood, that is more like me. I am a confident and steady person who can give the clear message: I am not scared by you mentioning death.*

The personal quality of feeling safe is described as being about a personal feeling, but also about a quality that is conveyed to others in a crisis situation. It is a personal quality that forms a basis in them, but it is also described as being learned during the evolving of personal history. The feeling and communication of safety is crucial in a work role that often is unpredictable and insecure for the professional and for the patient.

Being creative was presented as an important personal quality by a few of the participants. It was described as using an artistic variable, being safe enough in one's own knowledge and experience to be able to do things that might be seen as unprofessional. Stepping out of the box that represents traditional treatment, doing those small things that were not traditional treatment but which had a therapeutic effect. Three participants connected the creativity aspect to such personal qualities as independence and self-reliance. These are important qualities for their professional life in the team. 

Other personal qualities that were described as important were the ability to withstand a high level of stress oneself, as well as recognizing a high level of stress in the patient, being able to stand back and not rush into a situation, being dedicated to and interested in the job, wishing and liking to talk to people, and humor at work.

The personal characteristics described here are all abilities that form the individual team members into persons that are fitted for the specific kind of work that is done in a CRHT team. They are qualities describing social abilities as being good at establishing deep emotional relations quickly, being safe oneself as well as making others safe, and the ability to be creative. These are all basic personal qualities forming the individuals. They are part of the team members' personal histories in the way that personal characteristics form how choices and decisions are made. The conscious understanding of who they are and what they are good at has in many of these stories given a direction to steer by when choosing a professional role. 

### 3.2. The Relationship between Specific Personal Interests and the Work Role

The personal history was connected to the professional role through the personal interests of the participants. The personal interests are part of them that have evolved during their personal histories, and have taken them in the direction of working in a CRHT team. All participants described how they had specific personal interests that they related to their professional role. The themes seemed to evolve with their profession stories and had a connection to their present work. Themes from their personal histories emphasized by the participants as being important to their professional role in the CRHT team were action orientation, network orientation, finding meaning and ethics in work, and seeing the whole person.

Nine of the participants described a wish to be action oriented. For some of them this was described as an important aspect of their professional role. This wish was illustrated by explaining how they had repeatedly held jobs that were action-oriented like crisis or emergency units during their career. Others connected this to personal qualities such as their own need for action and something to happen.

*I loved being responsible for the emergency calls when working on the children's unit. To be called in for a really sick child, followed by the parents, that was emergency psychiatry as well as helping the kid. I love that. I really do. It excites me. I would like to do more of that. You know, a great adrenalin kick.*

An action-oriented way of working was described as quickly moving in and out of a case. Taking a case, assessing it, retrieving a solution, and closing the case in a short time, demands that the individual is good at building relations quickly. It also required a high degree of knowledge and professionalism.

*I like fast relationships. I like blue lights. And also the level of knowledge, I am swimming in professionalism. The joy of being surrounded by so many competent professionals every day is different from anything I have experienced in my professional life before.*

There was also an aspect of unpredictability in this action orientation. Four participants describe how they were attracted by the unpredictability and excitement of their everyday work life. Each case was different and exciting; there were changes all the time. The participants liked the feeling of daily new experiences and challenges which the job brought.

The personal interest for the action orientation is closely linked to the professional role in the CRHT team. The everyday work in the team demands that team members move in and out of cases quickly, that they have a high tolerance for unpredictability as every new appointment is about meeting a patient in an acute mental health crisis. And meetings with patients might often be chaotic and dramatic. The professional role in the team demands personal that can endure this kind of action in their work role over time. 

The wish to follow a network-oriented pattern of work was described by six participants as meeting the private network surrounding the patient, and by ten participants as working in the patient's home. Those focusing on the network were concerned about the family surrounding the patient, if there were children involved, and what the relations between the patient and the network were like. When talking about working with the network, there was a dimension of understanding how the network suffered, while also understanding the importance of including the network in the treatment. These participants had previously experienced working with children, adolescents, or families of mental health patients in different ways. Their previous experiences were linked to their perception of the importance of working with the network to get a broader understanding and to help the patient in the best possible way.

Home treatment was a key subject in ten of the interviews. The participants found that working in the service user's home demanded another level of respect and balanced out the power imbalance between the patient and the professional.

*When I go into a patient's home, something happens regarding symbolism. I am a guest, I take my shoes off, and I sit nicely on the couch. There is something different going on from what happens in in-patient care.*

The participants' understanding of this form of treatment was explicitly connected to their previous work experience. Episodes in their previous personal history had given these team members experiences in how patients were met in in-patient care and how they were met in home treatment. They had experienced that they themselves, as well as other health professionals acted in another way, treated the patient as a person differently when doing home treatment than what they did in in-patient care. The symbolism in visiting in the patients' home affected how they saw themselves and the patient, and evened out the power balance in the relationship. This understanding from their personal history steered them in a direction of having a professional role where they could do home treatment as much as possible.

Five of the participants described believing in what they did as an important aspect of their professional role. Their personal experiences of believing in what they did were connected to experiences in their professional role. Seeing how patients were treated and how this affects them was described as triggering positive or negative reactions. This resulted in a wish either to do the same or the opposite in terms of treatment as well as how to approach the patients. 

Ethics were described by ten of the participants as a dimension of respecting the patient but also as an aspect of treatment methods. The respect for the patient was important. Good experiences were described as seeing the patient as a customer; approaching him in a proper way, showing respect and being equals. There were also experiences of approaching the patients without respect or even with disrespect.

*The workplace was adding up to a lack of respect for the patients. I could not be a part of that.*

There was another dimension of ethics in how the participants perceived the treatment methods used on the patients. A few participants commented that there was no real treatment for the patients' mental health problems when they were submitted to mental health care. The lack of treatment was described as an ethical dilemma for the professionals. Another aspect was how the participants believed in certain treatment methods and not in others. Three participants described how treating patients without believing in the method used was an ethical dilemma for them. This experience was related to a wish to work with methods that one believed in, and was described as leading towards the professional life they had at the moment, as clear connections were made between previous work experiences and current professional role.

*I was asked to start working in the CRHT team. I believe this is the right way to work. Seek out people at home where they are and treat them there. Instead of moving the patients around from place to place and finally sending them home. So this was perfect for me.*

For seven of the participants, there was a wish to address the whole person in the helping relationship. These participants emphasized a wish to work with the whole person as a mental, physical, and social individual. One of these participants described understanding the connection between psyche and soma from a previous working experience in a somatic unit. Another participant described the experience of seeing the patient as a social individual.

*There is something important about the sense of belonging and attachment. To be seen by someone and that somebody is looking out for you. Someone asks if you are ok, and maybe even sees that you are feeling bad without the need to ask. Sometimes that is all that is needed to see a patient blossom. *

The participants saw mental, physical, and social aspects of the patient as related to each other. They had a perception that all of these aspects needed to be treated to help the patient heal. 

The personal interests as described by the participants are part of their personal history. From early years, through growing up, studies, and work their personal interests have evolved and became clear and conscious to them. The participants' individual personal interests as having strong feelings for action, network, meaning, ethics or seeing a person as a whole is part of their personal history, based in experiences in their lives. When talking about these interests they link them to choices that has given direction to how they ended up in the professional role in the CRHT team were they are at the moment.

### 3.3. Formative Work Experiences on the Way to Be a CRHT Team Member

Formative work experiences are, together with the personal characteristics and the personal interests, an important part of the personal history that the participants linked strongly to the professional role. Twelve participants talked about personal experiences that had been important for their professional role. These ranged from experiences in work-related situations to experiences related to private or family life. Common to these experiences were that they were described as having a great impact on how the professional role evolved.

Five participants described how early working experiences with psychotic, suicidal, and violent patients stimulated an interest in working with patients with severe mental health problems. One of them described striving for these types of jobs from the start of her career, while another talks about how a dramatic violent episode at work imparted an understanding of the need to learn more about severe mental health problems and provoked a choice to start nursing training. 

One of these participants described her choice to work in an emergency response center at the beginning of their career, even before commencing her studies. Serving a telephone line, part of the job was to guide patients at home with mental and physical problems while waiting for the ambulance to arrive. For patients living in sparsely populated areas, the telephone support could last for hours. This job was combined with driving an ambulance and taxi driving.

*You experience a lot of different things. I did. I have been in the strangest situations in those jobs. Once I had a big knife at my throat. I think these early experiences have helped me, in that it takes a lot for me to get stressed.*

One participant described how early work experience in a nursing home raised her consciousness of how patients were treated. When observing how a patient's opinions were ignored, the participant identified with the patient, recognizing how one's own opinions as a young colleague were also ignored. The focus was on the ethical aspects of treatment.

*I felt I was compromising on my work ethics a lot of the time. There was no time to talk to the patients, which I saw as a very important part of my job when choosing this profession. Instead our focus was on how fast we could get everybody to bed and which patient could go first.*

One participant found that tough work situations suited her personality.

*I worked with severely ill patients; it was really tough and challenging physically but most of all psychologically. It made me discover that it suited me. It also made me realize the importance of taking care of my own mental health. There was not a lot of follow-up from the employer, but between colleagues we used to talk and help each other out.*

This participant also described the process of trying out different professions, but felt a personal identity in mental health services. Even when working in places that were not connected to mental health services, the participant finds herself taking the mental health perspective as part of every assessment. 

One participant described getting an eye-opener on the importance of mental health when working on a somatic ward.

*One morning there was a patient on the neurological ward with a really bad headache. She had a CT, but that showed nothing. So I was with the patient when the assisting medical doctor came to see her. The doctor started asking really simple open ended questions. That was like turning on a switch, and the patient started talking about all the responsibility and worries she had at home. After a while she stopped, looked at the doctor and said “Why am I telling you all this, I have never told anyone about this before”. Then she realized her headache had gone. It made me think about the questions the doctor asked, what they were, how she did it, how they helped the patient. It was like a kick to me. It made me curious. It was so interesting, it made me think “No, you have got to continue on with this. And then I applied for studies in mental health services”.*

One participant described how concrete episodes of meeting patients and colleagues in different mental health services had expanded her belief in certain ways of working, for example, home treatment and network orientation. The participant connected episodes from her personal history to an understanding of what was helpful for the patient. The episodes form the way of thinking and working, as well as steering the professional role towards areas of mental health services where the participant's own understanding is accepted and important. This may be an example of how the personal history and the professional role are closely linked together; elements from the personal history, here represented by a profound work experience, give direction to the professional role.

Important work-related experiences can be immediate, referring to one concrete episode such as the one above. They can also appear over a period of time. Three participants described how friends or colleges had introduced them to mental health and motivated them to start working with it. One of the participants had a family history of severe mental health problems, but did not think it would be possible to work within that area. Although having worked with mental health for the last ten years, the participant pointed out that she had only understood that she was good at it in the past two years. Another participant described how a friend told stories from work. The participant got interested and decided that this was an interesting career choice. During practice the participant was introduced to mental health services, found it both frightening and exciting, and decided to continue working with severe mental health problems. For some time the participant felt a lack of knowledge when encountering a difficult case, which led to more education related to mental health. The experience evolved over time. This participant also had an immediate experience that was described as important for his understanding and thoughts about work.

*I remember one episode that was an eye-opener for me. It is quite typical that when working and working and feeling that there are no changes in the patient, that the work is not leading anywhere, you get frustrated and irritated. There was this group supervision that really made me rethink things. If I want a change I have to change myself, I cannot change other people. I can choose to change my own thinking regarding a problem. I can choose to do something different in a given situation.*

One participant referred to an experience of how the personal history could be used to benefit the professional role. The participant lost a close relative when they both were young. When approaching families who had lost somebody, the participant recognized the feeling and knew the subject personally when talking about death. The participant's personal experience of death affected her approach with families experiencing the death of a close relative in their personal life. This understanding produced a reflection on how personal experiences contributed to growth in the professional role as well.

*I have become better at wondering. At the beginning of my career I was more certain about how things were supposed to be handled. This is supposed to be like this or like that if it is to be done correctly. I did not wonder much about anything, but now this has changed. I do not always have to be right any more. Others might also be right and that is ok.*

The formative work experiences are part of the personal history. While the personal characteristics and the personal interests are part of the individual that evolves from early years, the formative work experiences are the part of the personal history that takes place in a work-related context. By describing experiences of working with psychotic, suicidal, and violent patients, becoming concerned about ethics, realizing how specific work situations suites one's own personality, and being drawn towards the mental health perspective the participants shows how specific experiences in a work-related context was important for choices they made regarding their professional role. Work-related experiences have given them an understanding of having a high tolerance for work with patient with severe psychotic, violent, or suicidal behavior. They have an understanding that working like what they do in a CRHT team suites their personality. And they have a perception that certain ways of thinking and working gives the results they are looking for. These experiences have given them individually preferences for and an understanding of the importance of the work in a CRHT team: doing crisis resolution as home treatment for patients in an acute mental health crisis. 

### 3.4. What It Meant to Relate to a Family Member with Mental Health Problems

Family related experiences have an impact on an individual's personal history. Having experiences of a family member striving with mental health problems as part of one's personal history are linked strongly to the professional role. One participant talked about how elements from her family history and her own life experiences affected her professional role. The participant's grandmother had a severe mental health diagnosis, and this knowledge made the participant think that working in the mental health service might be too hard and would make no difference to service users. This view changed as the participant's career evolved. The personal history became a part of the professional role in terms of understanding how it was to be a child and a relative of a service user, when to start working with the mental health service, rethinking one's own personal story and understanding connections between one's personal history and professional role.

*My mom was 9 years old the first time grandma had an ECT. This was in the 50's. Grandma did not remember her daughters after the ECT. My mom had quite a traumatized childhood. I can feel how my mother's experiences have given me a commitment to work with children of parents with mental health problems.*

Due to knowledge of her mother's story and the impact seen on her, the participant had an interest in and awareness of the children in families where one of the parents suffered from mental health problems. The participant described a clear understanding of a connection between her personal family history and her interest in children and the network of service users.

*I think I have a special interest in children and the network of service users. This is based on my own experiences with my grandma, and particularly my mother's experience of having a mother with mental health problems. Both of us have personal experiences of how that really feels.*

The participant emphasized the importance of knowing one's own personal history and at the same time being aware of how it affected one's professional role. There was an awareness of being close to one's personal history and at the same time managing to maintain a distance that made reflection on choices in one's professional role possible.

*I have personal experience of mental health problems in the family through my grandmother and I also have personal experience of being a single parent. When I meet single parents with children amongst the service users I can feel it inside of me. I want to fight for those children. I know that those are the times when I need to back off and take a minute to think: is this about my own experiences or about the service user I am meeting right now? Maybe this is why I needed to do other things before I started working in mental health. I needed to create some distance from my own personal story and gain the ability to reflect up on it.*

*I needed to find the words for my own personal story, my own experiences, to be able to put them into words, go through it, reflect up on it and make it a conscious part of myself. So that I was able to say: this is my story and this is what it has done to me. I think I would have acted differently than I do today if I had not done this reflecting upon my own story. Then it would have been more difficult in certain situations to know if I were acting on what the service users need, or on my own feelings based on my personal story.*

The time and effort spent in understanding and making the personal history a part of oneself was important for the connection between the participant's personal history and professional role. Without doing this the personal history could have been problematic to handle when approaching the service users. The participant described how her personal history could have interrupted her understanding of the service users' perspective. But understanding and being aware of the personal history provided the advantage of extended knowledge based on personal experiences. Obtaining closeness to and at the same time distance from personal experiences was crucial in order to connect the personal history to the professional role, such that the latter could benefit from the former. The participant saw and described clear connections between the personal history and the professional role, and how the one impacted on the other.

This participant's description of her personal history and professional role gives a good illustration of how the personal history stretches from early childhood, through life and into the professional role. Experiences of the personal history from childhood and adult life become conscious, are reflected upon, and are brought into the professional role as elements that contribute to the role. 

### 3.5. What It Meant to Relate to Family Members Working in Mental Health Services

Family members working in mental health service is part of the personal history that participants link to the professional role. Four participants discussed how family members working with mental health services or with people in difficult life situations had been an inspiration for their own choices, and had made this particular choice of career easy through familiarity with it. One of them had several family members working in the mental health service. Both the participant's grandparents worked with service users who had severe mental illnesses. As a child the participant remembered accompanying their parents to visit a friend on a psychiatric ward. The participant described how all these experiences made mental health services and severe mental health problems familiar, and how they influenced her personal history.

*I have always had this ballast from my grandparents, that the people they worked with were very sick people. My grandfather was a police officer and worked as a guard at the hospital. He was with the patients when they were very ill. My grandfather was very calm, so he managed to calm the patients down as well. The attitudes I have learned from them have had a great influence on me.*

These experiences of family members working in mental health services are part of the personal history that is described as having an impact on the participants' professional role. Experiencing family members working with service users with severe mental health problems gives the participant an insight that seems to result in a feeling of safety and familiarity that contributes to a higher tolerance in the professional role when working in the CRHT team. As the team works with service users with an acute mental health crisis staying at home, often meeting stressful and overwhelming situations, a higher tolerance might be an important quality for these participants. The personal history, here represented by experiences of family members working in mental health services, provides necessary and important qualities to the professional role in a CRHT team.

## 4. Discussion

Based on the findings, there are two aspects to be discussed further. First, the participants do experience a relationship between their personal histories and professional roles. They create narratives based on individual experiences and cultural context, their personal histories, which give meaning to their actions and choices, throughout their carrier and in their professional role [11, 31]. Secondly, three main themes emerge from the interviews that elucidate how CRHT team members experience the relationship between their personal histories and professional roles. The themes are experiences of the relationship between oneself as a person and one's work role, work experiences, and family experiences. These are elements that may be seen as developing through their life stories and construction of identity as described by McAdams [10] and Schütz and Wagner [30]. The elements are experienced as connected to the work in the area of CRHT.

### 4.1. Experiences of the Relationship between the Participants' Personal Histories and Professional Roles: Meaning, Life Stories, and Identity

The participants make connections between their personal history and professional roles. There are differences in what themes they emphasize, in what connections they make and to what degree they explicitly make the connection. For some participants the story is told as a continuous narrative where each part is based on the previous one, and relationships are implicit. In other stories specific connections are made explicitly. Polkinghorne [31] describes how the narrative of a person's life is formed and also develops during life. The narrative of life is written and rewritten as a history, as life goes by and is constantly changing [31]. The purpose is to create a meaning to life. The starting point of our interviews was whether and how there were any connections between the personal history and professional role as described by CRHT team members. The interviews show how most of the participants describe links between the two, and also gives some meaning to how the personal history has affected the professional role. 

Connections are made between experiences of the relationship between oneself as a person and the work role, work experiences, and family experiences. Elements from the relationship between themselves as persons and their work role, profound experiences in their work context and their family stories contribute to how they understand and explain that they are working in a profession like the CRHT team. There are differences in how they emphasize their experiences. Some participants emphasize their family experiences, while it is more common in this sample to highlight the relationship between themselves as persons and their work role. Bruner explains how meaning is constructed by the individual but also by the culture through a shared social history. This allows an individual continuity [11, 32]. 

Family experiences, in the form of knowledge of mental health problems through family members who have been ill or through relatives working in mental health, are described as experiences that give knowledge in the form of a deeper understanding, familiarity, and recognition of severe mental health crisis. For these participants, this is experienced as leading to a higher degree of safety when encountering severe mental health problems. 

The element of safety is resumed when the participants talk about the relationship between themselves as people and their work roles. The question of being safe oneself in approaching severe mental health problems at work is connected to the knowledge, understanding, recognition, and familiarity that come from the participants' family stories. Family experiences are described as giving the participants personal ballast they carry with them when approaching patients in severe mental health crisis. It gives the participant a feeling of safety in the situation, and it helps them convey safety to the patients. This reflects both the individual and the cultural aspects of Bruner [11].

To look for meaning, seek an ethical way of working and meet the patients are all described as part of the relationship between oneself as a person and the work role. The participants describe this as a part of themselves that evolved and is integral to them as individuals. When talking about profound experiences in a work context, these aspects appear again in their early work experiences as well as in the profound experiences of work, both in terms of eye-opener experiences and those that evolve over time, illustrating the evolution in narration as described by Polkinghorne [31].

Hearing the participants' experiences of and the relationship between their personal histories and professional roles, there seem to be remarkable connections between their family stories and how they describe themselves as individuals, and their professional stories. There are different kinds of relationships, some more explicit, others more implicit, but most of the stories impart meaning and some form of understanding to how things unfolded as they did regarding the professional stories. The participants refer to relationships that they can see. Only rarely are comments made regarding choices based on coincidences. Coincidences are mentioned in reference to, for example, applying for certain jobs, but then again coincidences are described as leading to meaningful and important experiences. The participants give meaning and direction to their experiences. 

What the participants share of their personal histories and professional role is what Schütz calls the life-world [30]. Schütz looks at the life-world from three dimensions: the individual person's “natural attitude,” the long chain of life experiences in the individual's life, and the “store of experience” that each individual possesses [30]. The experiences of the participants as described in the material are in line with all three of these dimensions. The personal characteristics described by the participants can be understood as one element of what Schütz calls the individual person's “natural attitude.” The development of individual interests, family experiences and work experiences, is all part of the long chain of life experiences that contributes to the “store of experiences.” There are clear parallels between the data and the dimensions describing Schütz's concept of the life-world.

Most of the participants' stories include elements of their own family story, dilemmas that reach turning points and references to how or why working in a CRHT team is in line with their choices. This way of telling a story of their personal history and professional role is consistent with how McAdams [10] models that biography is structured in Western culture. It is suggested that biographies start with family experiences, expanding and tracing later problems to earlier conflicts, incorporating turning points that mark changes, and continue into a story of progress or decline [10]. The stories from the participants reflect this way of telling a biography, with elements of all these aspects in their stories. The turning points are shown through the relationship between oneself as a person and the work role or through work experiences. The progress is connected to the participants' work experiences and how they now work in the CRHT team, describing how and why this work is preferred and a relevant career choice at the moment. The participants often justify their work in the CRHT team by showing that it is meaningful, interesting, and give them room for creativity in their work. 

When telling their biography the participants construct an identity. The shaping of identity is an ongoing process throughout life. Identity can be understood as the staging or arranging of the self. These are not consistent, but are subject to how the participants are working at their narratives over time [10]. 

The three themes presented in the findings have three different characteristics. Family experiences can be seen as a frame for the informants as it possesses referential properties. The relationship between oneself as a person and one's work role merges the person with the work role. These two themes constitute the personal biographic experiences of the individuals. Selectively the team members extract what is meaningful based on these experiences. The work experiences can be seen as characterized by a force that pushes the individual in certain directions. Based on these experiences the informants create professional meanings. Their stories are shaping and reshaping in the way they practice. The practice is ongoing and also changes.

As described by the team members in the CRHT team, these parts of personal history all to different degrees contribute to giving direction and meaning to working in the team. The professional role is very rarely described as coincidently, and rater as a result of interaction between parts of the personal history. 

### 4.2. Reflexivity on the Researchers' Role in Interviewing and Analysis

It is relevant to examine how the researchers' theoretical orientation might have interfered with the interviewing and analysis. The hermeneutic-phenomenological approach and the use of reflexivity offer an opportunity to explore how to understand and emphasize the data [24, 26]. The interviews were done within a specific context, consisting of a concrete group of researchers and participants. The two researchers who conducted the interviews have a relationship to home treatment and network orientation in CRHT by working with national training for CRHT teams, although to different degrees. These two researchers were known to the team even before starting the research period, as was their understanding and positioning regarding work methods for CRHT teams and the national public documents on this subject. This may have had an effect on the participants when choosing what to emphasize in their stories. Other researchers, who did not know the team in advance, may have received other answers then the known researchers did [20]. On the other hand, the researchers did not raise questions regarding the participants' theoretical orientation in CRHT work. The participants pointed to a variety of different experiences that they perceived as important from their personal experience. These do not seem to be characterized as what the researchers may have expected to hear. The researchers focus was on the participants' personal history and professional role, and not on whether the work was in line with the national public documents.

The cultural horizon the researchers shared with the participants was that national public documents state that the CRHT teams are expected to be multidisciplinary, to do rapid assessment, home treatment in crisis resolution and to be network oriented [33]. Some of these elements arise as important aspects from the interviews. The themes are introduced by the participants and followed up during the interviews by the researchers. They are thereby given an important role in how the participants describe their experiences. This might have been different in another cultural setting, where these subjects were not known to be relevant. In the present cultural setting they are relevant and emerge as subjects that are described as important. 

### 4.3. Implications for Research on Experiences of the Relationship between Personal Histories and Professional Roles in Mental Health Care

Listening to the participants reveals connections and meaning in their stories. The participants' experiences of life give meaning to their personal histories and professional roles. Through their stories they offer an understanding of their experiences and of how they are related in a meaningful way to their professional stories of today. Bruner [11] describe meaning as defined by the individual and the culture the person belongs to. For the self to create a meaning it is also dependent on the practice which it is a part of. The participants' personal histories and work roles are individual experiences, and they are given meaning in the cultural context of one particular CRHT team as well as within a national cultural context. Both Polkinghorne [31] and McAdams [10] describes how the narrative of the individual is an ongoing developing process which the individual is rewriting as life progresses. The participants are in the middle of their lives, and their life experiences as they relate them are their understanding as they see it at the moment, enlightened by the cultural context.

Our findings explore how people working in CRHT experience the connection between their personal histories and professional roles and indicate that there is indeed a relationship between these. The participants give meaning to the experiences and the relationships explored. They indicate that their present work in a CRHT team is connected to the relation between oneself as a person and the work role, former work experiences, and family experiences. Working in the CRHT team might be seen as distinctly different from other services in mental health. According to the findings in this paper an implication for practice might be that working with crisis resolution in home treatment raises a need for professionals that are capable and willing to work in risky situations, assessing patients in crisis daily. Based on the knowledge of this background information, the participants are giving meaning to their personal histories and professional roles to give some understanding of the relationship between these. 

Through the personal histories and professional roles, layers of experiences are constructed. Episodes in the history are given meaning by the participants. 

For future research implications, it may be interesting to examine more closely experiences of personal histories and professional roles amongst CRHT team members and personnel working in different areas of mental health services—what their experiences are and how they write meaning into their personal histories and their professional roles.

### 4.4. Limitations

The interviews give a view into a wealth of experiences from the participants' lives. However, this paper is based on single interviews, a series of interviews with each participant would have given possibility to go into more detail of their life-stories, and highlight more connections between the personal self and the professional role. Comparing the experiences of participants from different contexts could also have given a greater richness to the material. The aim of this paper was to describe the team members' experiences and how they make connections between their personal histories and professional roles. The descriptions from the interviews give a rich variety of stories, but also some clear equality in the relations between the participants' personal and professional lives.

The interviews are conducted by two interviewers. There was an interview protocol set up and a common idea of how to conduct the interviews. However, differences in the interviews due to the personality of the interviewer are unavoidable. If the participants were switched between the interviewers, the interviews might have showed different sides of the participants' life stories [20].

## 5. Conclusion

The aim of this paper is to explore whether and how CRHT team members experience the relationship between their personal histories and professional roles. The participants emphasize that there is a relation between the two. They give meaning to the relationship between themselves as people and the work role, work experiences, and their family experiences when relating these experiences to their current work in a CRHT team. There are few studies exploring how the relationship between personal history and experiences affects the professional role. Specifically in mental health services, where the individual filling the professional role is inevitably a part of forming the role, this relationship is an interesting and important aspect of the work each individual performs. The relationship between professionals' personal histories and professional roles may have implications for further research, exploring more of how it affects different kinds of work roles within the mental health field. This may be especially important when it comes to new roles putting professionals into the kind of emotional and relational challenges inherent in being a CRHT member.

## Figures and Tables

**Table 1:** Illustration of the steps in data analyses.

Citation	Content unit	Meaning code	Conceptions and overall description
“I can be scared, but I cannot remember that that has ever happened when with a patient.”	Feeling safe in stressful situations	Feeling safe oneself	Characteristics of one self as a person
“I can see how younger colleagues talk to the doctor more often because they feel unsafe regarding the patients. That is a way that they can gain experience and confidence.”	Feeling of safety can be learned		

**Table 2:** Themes from the participants' personal histories that were linked to their professional roles.

Experiences related to the participant as an individual	Work-related experiences	Family-related experiences
(i) Personal qualities (ii) Personal interests	(i) Profound personal experiences in a work context	(i) Family members with mental health problems (ii) Family members working in mental health services
